# Reliability and Safety of Cross-Leg Free Latissmus Dorsi Muscle Flap in Reconstruction of Mutilating Leg Injuries Using End-to-Side Anastomosis

**DOI:** 10.1055/a-2126-7058

**Published:** 2023-10-05

**Authors:** Ahmed Gaber Abdelmegeed, Mahmoud A. Hifny, Tarek A. Abulezz, Samia Saied, Mohamed A. Ellabban, Mohamed Abdel-Al Abo-Saeda, Karam A. Allam, Mostafa Mamdoh Haredy, Ahmed S. Mazeed

**Affiliations:** 1 Department of Plastic and Reconstructive Surgery, Sohag University Hospital, Sohag, Egypt; 2 Department of Plastic Surgery, Faculty of Medicine, South Valley University, Qena, Egypt; 3 Plastic and Reconstructive Surgery Unit, Suez Canal University Hospitals and Medical School, Ismailia, Egypt

**Keywords:** cross-leg, end-to-side anastomoses, free latissimus dorsi flap, single-vessel leg, mutilating leg injuries

## Abstract

**Background** Free tissue transfer is considered the gold standard option for the reconstruction of distal leg defects. Free tissue transfer using recipient vessels in the contralateral leg (cross-leg bridge) is a potential option to supply the flap if there are no suitable recipient vessels in the injured leg. Most studies have described this technique using end-to-end anastomosis which sacrifices the main vessel in the uninjured leg. This study evaluated the use of a cross-leg free latissimus dorsi muscle flap for the reconstruction of defects in single-vessel legs, using end-to-side anastomosis to recipient vessels in the contralateral leg without sacrificing any vessel in the uninjured leg.

**Methods** This is a retrospective study that included 22 consecutive patients with soft tissue defects over the lower leg. All the reconstructed legs had a single artery as documented by CT angiography. All patients underwent cross-leg free latissimus dorsi muscle flap using end-to-side anastomosis to the posterior tibial vessels of the contralateral leg.

**Results** The age at surgery ranged from 12 to 31 years and the mean defect size was 86 cm ^2^ . Complete flap survival occurred in 20 cases (91%). One patient had total flap ischemia. Another patient had distal flap ischemia.

**Conclusion** Cross-leg free latissimus dorsi muscle flap is a reliable and safe technique for the reconstruction and salvage of mutilating leg injuries, especially in cases of leg injuries with a single artery. As far as preservation of the donor limb circulation is concerned, end-to-side anastomosis is a reasonable option as it maintains the continuity of the donor leg vessels.

## Introduction

Reconstruction of defects in the middle and lower thirds of the leg is challenging and requires innovative solutions. Microsurgical reconstruction with free tissue transfer has been considered the gold standard option for treating such defects, and this necessitates the presence of adequate recipient leg vessels. 1 2 Reconstruction is more complex if the patient has preexisting vascular compromise in the lower limb or when there is concomitant vascular injury. 3

If there are no reliable recipient blood vessels, the use of the contralateral leg vasculature is a potential option to supply the transferred tissues. Using cross-leg bridge free tissue transfer was first described by Taylor et al in 1979, and the principal concept of this technique is to anastomose the flap to intact vessels in the contralateral noninjured leg. This temporary vascular anastomosis supports the flap until it forms sufficient vascular connections with its edges and bed in the injured leg, then separation is performed. 4

A number of studies have reported successful reconstruction of leg defects with cross-leg free tissue transfer, utilizing muscle/musculocutaneous flaps such as latissimus dorsi, rectus abdominis, gracilis, and vastus lateralis muscles 5 6 7 8 9 10 11 ; cutaneous flaps such as anterolateral thigh and deep inferior epigastric perforator flaps 7 ; or osteocutaneous flaps such as deep circumflex iliac artery (DCIA) and fibular flaps. 7 11 12

Most of these previous studies used end-to-end anastomoses to the recipient vessel in the contralateral uninjured leg. The major drawback of end-to-end anastomoses is scarifying one of the main blood vessels of the uninjured limb. Therefore, several new reconstructive techniques have been introduced in order to maintain and preserve the integrity of the recipient arterial system of the uninjured leg. These techniques include redirecting the artery of the flap to the distal end of the recipient vessel after pedicle division 13 14 or preparing the arterial blood supply of the flap in a **Y** - or **T** -shape fashion and suturing it to the recipient vessel in a flow-through anastomosis fashion. 15 16

Although there are many studies which demonstrated the use of free tissue transfer with end-to-side anastomoses to recipient vessels in the same injured leg with the aim of preserving distal perfusion in patients with impaired vasculature, 17 18 19 there are no studies that reported the use of an end-to-side anastomosis for cross-leg free flaps as a method to maintain the integrity of the recipient vessel.

The purpose of this study is to present the outcomes of the use of cross-leg free latissimus dorsi muscle flap for reconstruction of defects in single-vessel legs, using end-to-side microvascular anastomosis to recipient vessels in the contralateral leg without sacrificing any of its vessels.

## Methods

We retrospectively reviewed 22 consecutive patients with traumatic soft tissue defects, in legs with a single artery as documented by CT angiography, who underwent cross-leg free latissimus dorsi muscle flap with end-to-side microvascular anastomosis to the posterior tibial artery of the contralateral leg. After the ethical approval of the study protocol, patients' data were collected from the patients' registry. The patients' demographic and clinical data included sex, age, type of injury, location of the injury, defect size, vascular status of injured legs, associated comorbidities, and time of flap separation, postoperative complications, and revision procedures.

### Surgical Technique

Under general anesthesia, sharp debridement is performed removing all necrotic or scarred tissues until reaching a healthy bleeding tissue bed. In the contralateral noninjured leg, the posterior tibial artery and one of its venae comitantes are dissected and prepared as recipient vessels. An inferiorly based skin flap, approximately 4 cm in width and length, is elevated to form the base of the bridge and protect the vascular anastomosis.

**Fig. 1:**
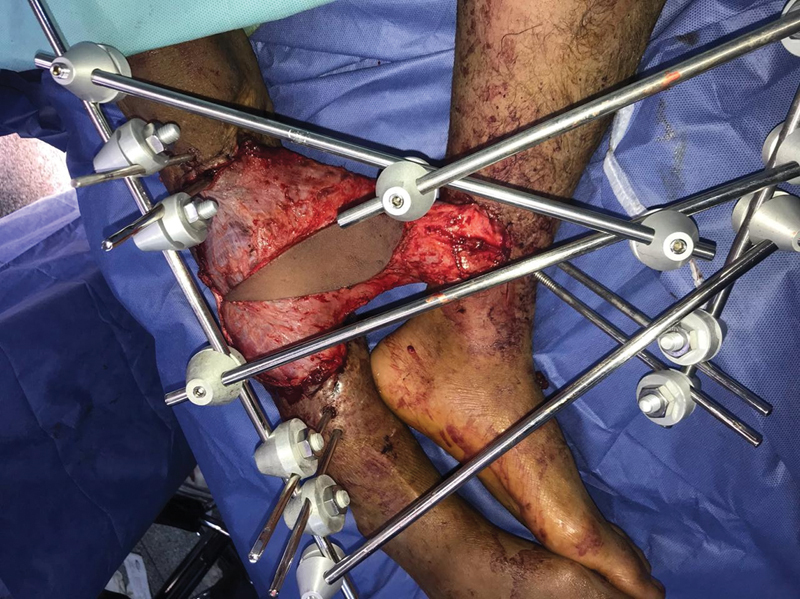
Intraoperative appearance of cross-leg bridge latissimus dorsi muscle flap with a skin paddle. The muscle is sutured to the edges of the defect in the injured leg allowing maximal interface between the flap and the recipient site. The two legs are fixed together using an external fixator.

With the patient in the lateral position, latissimus dorsi muscle flap is harvested with the dominant thoracodorsal pedicle. A small skin island is included with the muscle to facilitate flap monitoring. The muscle is then sutured to the healthy edges of the defect in the injured leg allowing maximal interface between the flap and the recipient site. The inferiorly based skin flap previously raised on the uninjured limb is turned and sutured to the opposing edge of the muscle flap to form the bridge ( Fig. 1 ).

The two legs are fixed together using an external fixator. End-to-side microvascular anastomosis is then performed between the thoracodorsal vessels and the posterior tibial artery and one of its venae comitantes on the contralateral uninjured leg. Patients are maintained on low-molecular weight heparin from the time of surgery till the patient is completely mobilized to protect against deep venous thrombosis. All patients stayed hospitalized until flap separation for monitoring of the flap and adequate wound care. The muscle is covered by a split-thickness skin graft either before the time of flap separation or during the flap separation procedure.

After 3 weeks postoperatively, the free flap pedicle was occluded with noncrushing clamps at the bedside and the flap vascularity status was assessed clinically using a needle prick test. If there is bright red blood bleeding in the distal part of the flap and no signs of insufficient flap circulation noted, we then conduct the second stage of flap separation and divide it from the recipient extremity. If flap circulation seems insufficient, we wait further 4 or 5 days and repeat that. After the removal of the external fixator and separation of the two legs, physiotherapy is started immediately to prevent joint stiffness.

## Results

The mean age at surgery was 21 years with a range from 12 to 31 years. Of the 22 patients, 19 were males and 3 were females. All defects were located in the leg. The defect was on the right leg in 14 patients and the left leg in 8 patients. The defect size ranged from 35 to 280 cm ^2^ (mean 86 cm ^2^ ). Follow-up ranged from 12 to 18 months, with an average of 15 months. The patients' demographic, clinical data, and vascular status of each patient are summarized in Table 1 . There were no associated comorbidities in our patients.

**Table 1:** Patients' data

Patient no.	Age (y)	Sex	Side	Etiology	Location of Injury	Vascular status (injured vessels)	Defect dimensions (cm ^2^ )	Follow-up period	Complications
1	12	M	Right	RTA	Lower third	ATA, PTA	8 × 5	16 mo	Partial skin graft loss
2	18	M	Left	RTA	Middle third	ATA, PTA	10 × 6	12 mo	Recipient site infection
3	27	M	Right	RTA	Middle third	PA, ATA	7 × 5	18 mo	–
4	16	M	Right	RTA	Middle and lower third	PA, PTA	12 × 6	15 mo	Total flap ischemia
5	31	M	Right	RTA	Lower third	ATA, PTA	8 × 6	12 mo	–
6	24	M	Left	RTA	Upper and middle third	ATA, PTA	13 × 6	14 mo	Partial skin graft loss
7	18	F	Right	RTA	Lower third	ATA, PTA	9 × 5	18 mo	–
8	19	M	Left	Train accident	Upper and middle third	ATA, PTA	15 × 7	14 mo	Distal flap ischemia Recipient site infection
9	15	M	Right	RTA	Middle third	PA, PTA	11 × 6	15 mo	–
10	19	M	Left	RTA	Middle third	ATA, PTA	12 × 5	12 mo	–
11	13	F	Left	RTA	Upper and middle third	ATA, PTA	18 × 11	18 mo	Partial skin graft loss
12	14	M	Right	RTA	Lower third	ATA, PTA	10 × 6	12 mo	
13	16	M	Right	RTA	Upper and middle third	PA, ATA	20 × 14	14 mo	Seroma Partial skin graft loss
14	27	M	Right	RTA	Middle and lower third	ATA, PTA	14 × 8	15 mo	–
15	29	M	Left	RTA	Upper third	PA, PTA	8 × 5	12 mo	–
16	19	M	Right	RTA	Middle and lower third	ATA, PTA	15 × 7	14 mo	Partial skin graft loss
17	25	M	Left	RTA	Middle third	PA, PTA	12 × 6	16 mo	–
18	20	F	Right	RTA	Middle third	ATA, PTA	13 × 7	12 mo	Recipient site infection
19	28	M	Right	RTA	Lower third	ATA, PTA	11 × 6	14 mo	–
20	22	M	Right	Firearm injury	Upper third	PA, PTA	8 × 7	18 mo	–
21	30	M	Left	RTA	Upper and middle third	PA, PTA	16 × 6	16 mo	Seroma
22	18	M	Right	RTA	Middle third	PA, ATA	14 × 7	14 mo	–

Abbreviations: ATA, anterior tibial artery; PA, peroneal artery; PTA, posterior tibial artery; RTA, road traffic accident.

**Fig. 2:**
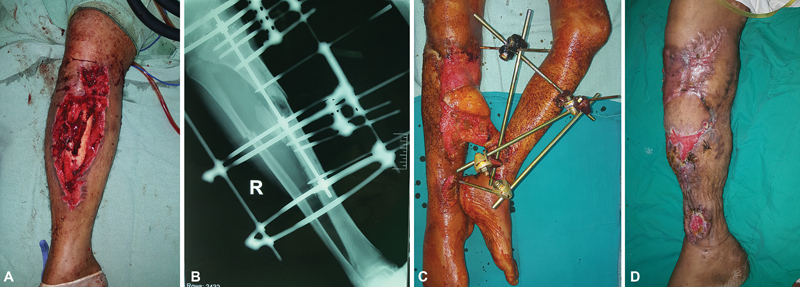
( **A** ) Preoperative appearance showing extensive soft tissue loss on the anterior and medial aspect of the right leg with exposed tibia and bone loss. ( **B** ) Radiograph showing fracture both bones of the leg. ( **C** ) Appearance 6 weeks after cross-leg bridge free latissimus dorsi muscle flap anastomosed to the posterior tibial vessels of the left leg. ( **D** ) Appearance 5 weeks after flap separation and split-thickness skin grafting. The flap is completely healed. There are small areas of skin graft loss which needed regrafting.

**Fig. 3:**
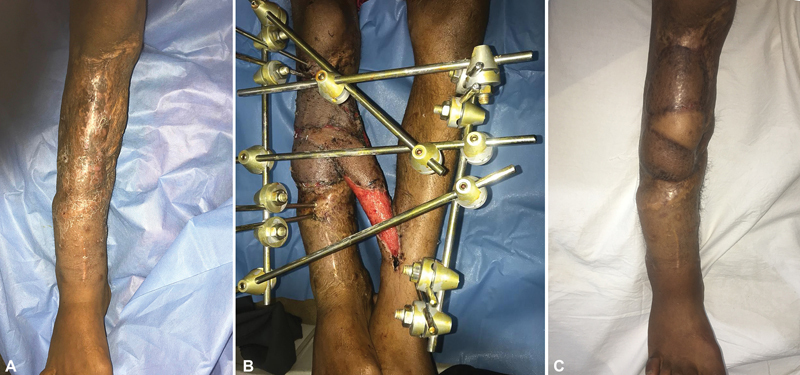
( **A** ) Preoperative appearance showing chronic unstable scar on the anterior aspect of the right leg and extensive scarring of the surrounding area, with a history of traumatic soft tissue loss due to road traffic accident and previous two skin grafting procedures. ( **B** ) Appearance 3 weeks after excision of the scarred tissues and coverage of the defect by cross-leg bridge free latissimus dorsi muscle flap anastomosed to the posterior tibial vessels of the left leg. Skin grafts are completely taken. ( **C** ) Appearance 16 months after flap separation showing complete healing and stable soft tissue coverage of the tibia.

All injured extremities were reconstructed with latissimus dorsi cross-leg free flaps as described in the surgical technique section. End-to-side anastomosis was performed to the contralateral posterior tibial artery in all cases to maintain distal blood flow. The second stage procedures were performed between 3 and 6 weeks. Figs. 2 and 3 show pre- and postoperative photographs.

Complete flap survival was reported in 20 cases (91%). One patient had total flap ischemia which was managed by debridement and negative pressure wound therapy for 3 weeks until the wound was clean with healthy granulation tissue, then a split-thickness skin graft was applied. Another patient had distal marginal flap ischemia that was managed by debridement, antibiotics, and wound care until healing by secondary intention occurred. Infection in the recipient site occurred in three patients and was treated conservatively by antibiotics and repeated wound dressings. Mild seroma in the flap donor site occurred in two patients and was managed by syringe aspiration for one time with no recurrence. All wounds in the contralateral leg healed uneventfully without complications apart from the anticipated scarring. Five patients needed regrafting of residual small exposed parts of the muscle flap due to partial skin graft loss ( Fig. 2D ). Although revision touch-up procedures were offered during follow-up, none of the patients requested further refinements.

## Discussion

Transposition of local tissue flaps (fasciocutaneous or muscle), if available, is the first choice to reconstruct defects of the leg. When there are no available local tissues for transposition, distant flaps and free flaps play a major role in these occasions. 2 20

Pedicled cross-leg flaps have been widely used for the reconstruction of leg and foot defects since 1854. 21 There has been no exact universal design for cross-leg flaps in the literature. Proximally based and transverse or oblique laterally based cross-leg flaps have been described. 21 22 With a better understanding of the anatomy of leg perforators, distally based and perforator-based flaps became more frequent. 23 24

The continuous improvement of the outcomes of microsurgical interventions has made free flap reconstruction a routine option in lower extremity reconstructive surgery. 19 Successful free flap reconstruction of leg defects requires optimal flap choice, in addition to selecting appropriate recipient vessels in the leg. 7 However, the lack of recipient vessels or the presence of single artery in the injured leg makes the free flap transfer a challenging and unsafe procedure. Many authors found that the rate of free flap failure increased when patent vessel numbers is decreased on the injured lower extremity. 25 26 Also, Khouri and Shaw demonstrated that the rate of anastomotic thrombosis doubled in single-vessel legs. 27 Although successful microvascular reconstruction of lower extremity defects using an end-to-side anastomosis has gained attention, especially in patients with mutilating limb injuries or impaired vasculature as it does not compromise distal perfusion, 19 28 an end-to-side anastomosis to the single dominant vessel, which supplies the ipsilateral limb, carries the risk of losing both the flap and foot. 29

Haddock et al described using the perforator-to-perforator anastomoses as another solution to avoid scarifying of the ipsilateral limb recipient vessel. 29 However, we believe that perforator-to-perforator anastomosis is technically demanding and is very difficult to apply in a limb with a wide zone of injury.

Therefore, it is necessary to use alternative options when there are no adjacent vessels available near the soft tissue limb defect and to perform the microvascular anastomosis outside the zone of injury. These include the use of vein grafts 30 as solutions to perfuse free flaps through anastomosis with a healthy proximal remaining blood vessel which is used as a recipient vessel on the ipsilateral extremity outside the zone of injury. Vein grafting requires another donor site, carries a risk of vein kinking, and increases the number of vascular anastomoses which subsequently raises the risk of thrombosis and free flap failure especially when using long vein grafts. 27 30 31 32 33 One study reported a fivefold increase in the incidence of anastomotic thrombosis when using vein grafts. 27

The use of cross-leg bridge free flaps is a relatively old surgical technique, firstly described by Taylor et al in 1979 to solve the problem of lack of vessels in the recipient leg using cross-leg free DCIA flap to reconstruct bony and soft tissue defect in the leg. 4 The technique is simply based on performing temporary anastomosis (end-to-end) between the vascular pedicle of the flap and an intact vessel of the contralateral leg that is transected then anastomosed to the flap and later divided as soon as adequate vascularization of the flap occurs from its edges and bed. 4 The cross-leg free flap is indicated when there is only a single nutrient artery supplying the extremity, when the neighboring vessels are damaged or not available and the use of vein grafting is not feasible or risky, and when the adjacent vessels were used in former free flap operation. 2

Cross-leg free flaps allow the transfer of the needed tissues including skin and muscle with or without bone using microvascular anastomoses performed away from the zone of injury to reconstruct challenging tissue defects with healthy vascularized tissue. 4 7 Since 1979, several studies have been published reporting successful reconstruction of leg defects using cross-leg free tissue transfer with end-to-end anastomosis. Some studies described using large muscle flaps such as latissimus dorsi which helped in the successful salvage of complicated leg defects 6 8 10 and in treatment of osteomyelitis. 5

Yamada et al applied the same technique in six patients using free rectus abdominis muscle flap anastomosed to vessels of the contralateral noninjured leg with a good outcome. 9 Townsend successfully used cross-leg free DCIA osteo-cutaneous flap in 10 cases to reconstruct leg defects with bone loss 6 to 12 cm. 12 Yu et al published a large series of 85 patients who underwent cross-leg free flap reconstruction utilizing a variety of flaps including latissimus dorsi myocutaneous, free fibula, free fibular osteocutaneous, and free iliac osteocutaneous flaps and showed an overall success rate of 95%. 11 Ozkan applied the same principle in 27 patients using different skin and muscle flaps including anterolateral thigh, latissimus dorsi muscle, gracilis muscle, vastus lateralis musculocutaneous, tensor fascia latae, and deep inferior epigastric perforator flaps, with 93% success rate. 7

Latissimus dorsi flap has many advantages as a cross-leg free flap in the reconstruction of complex leg defects as it can provide a sizable flap with a long pedicle and good vessel caliber. 31 Its large muscle component allows durable closure of complex wide defects. 6

The use of the posterior tibial vessels as cross-leg bridge recipient vessels has often been described in the literature as an end-to-end anastomosis as this allows easy positioning and safe anastomosis. 3 7 10 11 12 30 However, this entails sacrificing these vessels by transecting them at the time of anastomosis.

This problem has led to design several innovative techniques to maintain the integrity of the recipient arterial system. Akyurek et al described a new technique to restore the continuity of the recipient artery in cross-leg free latissimus dorsi flap procedure after end-to-end anastomoses. They reestablished the continuity of the posterior tibial artery at the time of flap separation by dissecting the thoracodorsal artery till its bifurcation in the muscle flap, transecting it, and reanastomosed to the distal ligated end of the posterior tibial artery. 13 14 However, this makes the flap separation another microsurgical procedure with prolonged hospitalization and possibly more complications. The flow-through pedicled free flap procedure is introduced not only to provide blood supply to the flap but also to preserve the integrity of the recipient vessel and distal leg circulation. 34 Topalan et al presented a cross-leg latissimus dorsi free flap procedure with the arterial thoracodorsal pedicle dissected in a Y-shaped for the flow-through continuity of the recipient artery. 15 Gencel et al executed the cross-leg free latissimus dorsi flap procedure where the thoracodorsal and circumflex scapular artery (or serratus branch, arterial pedicle) were fashioned as T-shaped and sutured to the contralateral posterior tibial artery in a flow-through anastomosis. 16 Although the flow-through free flap procedure is introduced to preserves the blood circulation in the healthy lower limb, it carries several disadvantages including the need to perform two anastomotic lines which increase the risk of postoperative vascular thrombosis. In addition, it is difficult to prepare a segment of Y-shaped or T-shaped arterial bifurcation during the harvest of free latissimus dorsi flap. 14 35

Yu et al suggested an alternative option to preserve integrity of the recipient vessel which is an end-to-side anastomoses to the contralateral limb in order to maintain continuity of the distal blood flow, although they performed the microvascular anastomoses of their cross-leg bridge free flaps series in an end-to-end fashion. 11 Several previous studies have reported the use of end-to-side anastomosis to recipient vessels in the same injured leg to preserve the leg vascular flow and reported no significant difference in the rate of flap complications between the outcome of end-to-end and end-to-side anastomoses in free flap reconstruction of lower limb defects. 19 28 However, to the best of our knowledge, there are no reported studies that described performing an end-to-side anastomosis in patients indicated for lower limb reconstruction using cross-leg free flaps as a method to preserve the integrity of the recipient vessel.

In the present study, we performed an end-to-side anastomosis of the thoracodorsal vascular pedicle of the free latissimus dorsi flap to the contralateral recipient posterior tibial artery while preserving the single vessel of the injured leg untouched and at the same time preserving the recipient vessels of the uninjured limb undisturbed after separation of the flap. This is the first case series which reported cross-leg free flap reconstruction using an end-to-side anastomosis to the posterior tibial vessels of the contralateral leg. We found that the use of this technique in 22 consecutive cases has resulted in an overall success rate of 91% which is comparable to the results of other previous studies describing cross-leg free flap using end-to-end or flow-through anastomosis. Furthermore, we believe that the end-to-side anastomosis technique, described in this study, have distinct advantages. The most obvious advantage is the prevention of reduction of the blood flow to the recipient extremity and protecting the continuity of the posterior tibial vessels of the noninjured leg while maintaining efficient free flap perfusion. This technique has also obviated the need to use long vein grafts or to prepare bifurcated thoracodorsal arterial pedicle that cannot be easily available for flow-through anastomosis. In addition, it seems that end-to-end anastomosis to a major artery causes problems such as temporary congestion or severe swelling of the free flaps which are induced by excessive inflow. These may be other advantages of using end-to-side anastomosis.

These findings suggest that cross-leg free flap using end-to-side anastomosis is an efficient and safe alternative for reconstruction of mutilating leg injuries with compromised vasculature, without sacrificing any vessel in the donor leg.

The limitation of this study is its retrospective design and being a case series without prospective data or a comparison control group using end-to-end or flow-through anastomosis which preserves the integrity of the contralateral recipient vessel. However, this series demonstrates that the end-to-side anastomosis to the contralateral recipient vessel is a viable option in cross-leg free flaps reconstruction supported by the high rate of flap survival and low complication rate.

In conclusion, cross-leg free latissimus dorsi muscle flap is a reliable and safe technique for the reconstruction and salvage of complex leg defects in mutilating leg injuries. It can provide a good reconstructive solution, especially in cases of leg injuries with a single artery, without increasing the risk of complications. As far as preservation of the donor limb circulation is concerned, cross-leg end-to-side anastomoses are a reasonable alternative as it maintains the continuity of the donor leg vessels for any possible future need.

## References

[1] Scott L L Baumeister S Lower extremity Philadelphia PA Saunders 2009 62 70

[2] Chen H El-Gammal T A Wei F Chen H Noordhoff M S Tang Y Cross-leg free flaps for difficult cases of leg defects: indications, pitfalls, and long-term results J Trauma 1997 43 03 486 491 9314312 10.1097/00005373-199709000-00016

[3] Yu L Tan J Cai L Repair of severe composite tissue defects in the lower leg using two different cross-leg free composite tissue flaps Ann Plast Surg 2012 68 01 83 87 21301302 10.1097/SAP.0b013e3181fe9351

[4] Taylor G I Townsend P Corlett R Superiority of the deep circumflex iliac vessels as the supply for free groin flaps. Clinical work Plast Reconstr Surg 1979 64 06 745 759 390575 10.1097/00006534-197912000-00001

[5] Ding S Y [Treatment of chronic osteomyelitis of leg with free latissimus dorsi musculocutaneous flap anastomosed to contralateral leg vessels] Chung Hua Cheng Hsing Shao Shang Wai Ko Tsa Chih 1993 9 02 106 107, 159 8221299

[6] Knobloch K Herold C Vogt P M Freier Latissimus-dorsi-Transfer zur Rekonstruktion von Weichteildefekten der unteren Extremität Oper Orthop Traumatol 2012 24 02 122 130 22446843 10.1007/s00064-011-0094-y

[7] Ozkan O Cinpolat A Bektas G Reconstruction of the lower extremity with cross-leg free flaps J Reconstr Microsurg Open 2016 1 01 12 18

[8] Sief K Shaker A Salvage of severely mutilated lower limb using cross-leg free latissimus dorsi flap Egypt J Plast Reconstr Surg 2010 34 01 17 22

[9] Yamada A Harii K Ueda K Asato H Tanaka H Versatility of a cross-leg free rectus abdominis flap for leg reconstruction under difficult and unfavorable conditions Plast Reconstr Surg 1995 95 07 1253 1257 7761513 10.1097/00006534-199506000-00017

[10] Yu Z J Tang C H Ho H G Cross-bridge free skin flap transfer: case report J Reconstr Microsurg 1985 1 04 309 311 4057171 10.1055/s-2007-1007091

[11] Yu Z J Zeng B F Huang Y C Application of the cross-bridge microvascular anastomosis when no recipient vessels are available for anastomosis: 85 cases Plast Reconstr Surg 2004 114 05 1099 1107 15457019 10.1097/01.prs.0000135331.08938.4a

[12] Townsend P L Indications and long-term assessment of 10 cases of cross-leg free DCIA flaps Ann Plast Surg 1987 19 03 225 233 3310809 10.1097/00000637-198709000-00008

[13] Akyürek M Safak T Ozkan O Keçik A Technique to re-establish continuity of the recipient artery after end-to-end anastomoses in cross-leg free flap procedure Ann Plast Surg 2002 49 04 430 433 12370652 10.1097/01.SAP.0000017989.80979.07

[14] Bali Z U Karatan B Tuluy Y Kececi Y Yoleri L Preserving the blood flow of the recipient artery in cross-leg free flap procedure for lower extremity reconstruction Int J Low Extrem Wounds 2020 19 03 255 261 32308079 10.1177/1534734620913414

[15] Topalan M A new and safer anastomosis technique in cross-leg free flap procedure using the dorsalis pedis arterial system Plast Reconstr Surg 2000 105 02 710 713 10697184 10.1097/00006534-200002000-00037

[16] Gencel E Eser C Kesiktas E Tabakan I Yavuz M A cross flow-through pedicle free latissimus dorsi flap for high voltage electrical burns Burns 2016 42 04 e55 e60 26559598 10.1016/j.burns.2015.10.014

[17] Fischer J P Wink J D Nelson J A A retrospective review of outcomes and flap selection in free tissue transfers for complex lower extremity reconstruction J Reconstr Microsurg 2013 29 06 407 416 23599213 10.1055/s-0033-1343952

[18] Pu L L A comprehensive approach to lower extremity free-tissue transfer Plast Reconstr Surg Glob Open 2017 5 02 e1228 28280670 10.1097/GOX.0000000000001228 PMC5340485

[19] Broer P N Moellhoff N Mayer J M Heidekrueger P I Ninkovic M Ehrl D Comparison of outcomes of end-to-end versus end-to-side anastomoses in lower extremity free flap reconstructions J Reconstr Microsurg 2020 36 06 432 437 32222958 10.1055/s-0040-1702156

[20] Zhang G L Chen K M Zhang J H Wang S Y Repair of a large soft tissue defect in the leg with cross-leg bridge free transfer of a latissimus dorsi myocutaneous flap: a case report Chin J Traumatol 2012 15 06 373 375 23186931

[21] Morris A M Buchan A C The place of the cross-leg flap in reconstructive surgery of the lower leg and foot: a review of 165 cases Br J Plast Surg 1978 31 02 138 142 346125 10.1016/s0007-1226(78)90064-4

[22] Barclay T L Sharpe D T Chisholm E M Cross-leg fasciocutaneous flaps Plast Reconstr Surg 1983 72 06 843 847 6139833 10.1097/00006534-198312000-00021

[23] Abdelmegeed A G Abulezz T A Abo-Saeda M A Allam K A Perforator-based pedicled cross-leg flaps in pediatric patients: a new idea to increase flap reach Ann Plast Surg 2021 86 05 568 572 32826434 10.1097/SAP.0000000000002523

[24] Hifny M A Tohamy A MA Rabie O Ali A AA Propeller perforator flaps for coverage of soft tissue defects in the middle and distal lower extremities Ann Chir Plast Esthet 2020 65 01 54 60 31477322 10.1016/j.anplas.2019.04.002

[25] Ricci J A Abdou S A Stranix J T Reconstruction of Gustilo type IIIC injuries of the lower extremity Plast Reconstr Surg 2019 144 04 982 987 31568316 10.1097/PRS.0000000000006063

[26] Arnež Z M Papa G Ramella V Novati F C Ahcan U Stocco C Limb and flap salvage in Gustilo IIIC injuries treated by vascular repair and emergency free flap transfer J Reconstr Microsurg 2017 33(S 01):S03 S07 28985632 10.1055/s-0037-1603737

[27] Khouri R K Shaw W W Reconstruction of the lower extremity with microvascular free flaps: a 10-year experience with 304 consecutive cases J Trauma 1989 29 08 1086 1094 2668545 10.1097/00005373-198908000-00005

[28] Cho E H Garcia R M Blau J Microvascular anastomoses using end-to-end versus end-to-side technique in lower extremity free tissue transfer J Reconstr Microsurg 2016 32 02 114 120 26322491 10.1055/s-0035-1563397

[29] Haddock N Garfein E S Reformat D Hecht E Levine J Saadeh P Perforator vessel recipient options in the lower extremity: an anatomically based approach to safer limb salvage J Reconstr Microsurg 2010 26 07 461 469 20464654 10.1055/s-0030-1254230

[30] Bayramiçli M Tetik C Sönmez A Gürünlüoğlu R Baltaci F Reliability of primary vein grafts in lower extremity free tissue transfers Ann Plast Surg 2002 48 01 21 29 11773726 10.1097/00000637-200201000-00003

[31] Luo S Gao J Luo J A case report of cross-leg free latissimus dorsi myocutaneous flap transplantation E J Plastic Surg 1999 22329 330

[32] Buncke H J Alpert B Shah K G Microvascular grafting Clin Plast Surg 1978 5 02 185 194 679603

[33] Germann G Steinau H U The clinical reliability of vein grafts in free-flap transfer J Reconstr Microsurg 1996 12 01 11 17 8618220 10.1055/s-2007-1006446

[34] Bullocks J Naik B Lee E Hollier L Jr Flow-through flaps: a review of current knowledge and a novel classification system Microsurgery 2006 26 06 439 449 16924625 10.1002/micr.20268

[35] Zhou H X He L Yin D Modified donor blood flow-preserved cross-leg anterolateral thigh flap procedure for complex lower extremity reconstruction J Orthop Surg Res 2022 17 01 262 35549724 10.1186/s13018-022-03155-9 PMC9097098

